# A comprehensive two-sample Mendelian randomization analysis of trigeminal neuralgia and modifiable risk factors

**DOI:** 10.3389/fneur.2023.1292958

**Published:** 2023-11-22

**Authors:** Xingrong Wei, Hao Zhou, Shuguang Zhang, Xueqian Hu, Zhenqin Wei, Yang Li

**Affiliations:** ^1^Department of Neurosurgery, The First Affiliated Hospital of Dalian Medical University, Dalian, China; ^2^Department of Graduate School, Dalian Medical University, Dalian, China

**Keywords:** trigeminal neuralgia, modifiable risk factors, Mendelian randomization, public health, epidemiology

## Abstract

**Objective:**

To conduct a comprehensive search and causality study of potential modifiable risk factors for trigeminal neuralgia. To provide new ideas for subsequent treatment and management of patients with trigeminal neuralgia.

**Methods:**

Data were obtained from large GWAS databases and then analyzed by Mendelian randomization analysis. The causal relationship between 36 potentially modifiable risk factors and trigeminal neuralgia was explored based on the results of the inverse variance weighting method(IVW). *p* < 0.05 was considered statistically significant.

**Results:**

Years of schooling [OR (95%CI), 0.59(0.42–0.84), *p* = 0.003] to be a significant protective factor. Anxiety disorders [OR (95%CI), 1.62(1.05–2.48), *p* = 0.028], Depression [OR (95%CI), 1.53(1.03–2.28), *p* = 0.035] and Autoimmune [OR (95%CI), 1.16(1.01–1.32), *p* = 0.033] were significant risk factors. Sleep duration [OR (95%CI), 0.43(0.18–1.01), *p* = 0.051] was a close protective factor. Body mass index [OR (95%CI), 1.24(0.98–1.57), *p* = 0.077] was a close risk factor.

**Conclusion:**

Mendelian randomization analysis shows Years of schooling and Sleep duration as protective factors. Anxiety disorders, Depression, Autoimmune, and Body mass index are risk factors. This will help in the research of diagnosis, treatment, and mechanism of trigeminal neuralgia. And reduce the prevalence of trigeminal neuralgia through positive psychological and lifestyle interventions.

## 1 Introduction

Trigeminal neuralgia is a neurological disorder that involves one or more sensory distribution areas of the facial trigeminal nerve. The incidence rate is 12.6 per 100,000, more women than men (1, 2). Typically characterized by paroxysmal, sharp, electric shock or cutting pain, it has been described as “the most intense pain ever experienced by mankind.” The pain can be triggered by everyday actions such as talking, eating, washing your face or brushing your teeth, and touching trigger points in the mouth, nose, or eyebrows (3, 4). The course of the disease often lasts for years, with symptoms getting progressively worse. Episodes range from intermittent to constant pain. The frequency of attacks becomes more frequent and the pain gradually worsens. During this process, the patient’s pain is intolerable, and it is difficult to sleep or eat, which seriously affects normal life and mental health. The causes of trigeminal neuralgia can be categorized into two main groups: primary and secondary. Primary trigeminal neuralgia is a type of trigeminal neuralgia in which no obvious abnormality can be detected on imaging tests, including CT, or MRI. The cause of the disease is not known. Secondary trigeminal neuralgia refers to the presence of clear organic lesions in the course of the trigeminal nerve. Although microvascular compression of the trigeminal root is the main cause of trigeminal neuralgia and can be demonstrated by imaging, pain caused by such compression is still classified as primary trigeminal neuralgia. However, such compression does not always lead to trigeminal neuralgia. The pathogenesis of trigeminal neuralgia, whether primary or secondary, maybe multidimensional (5).

The treatment of trigeminal neuralgia has shown a state of multidisciplinary intervention. The main methods of treatment include medication (carbamazepine), interventional procedures (balloon compression and radiofrequency thermocoagulation), and surgical treatment (MVD) (5, 6). However, the pathological and molecular mechanisms of trigeminal neuralgia are not fully understood (7, 8). Although various treatments have achieved some degree of efficacy, they are not satisfactory overall. In the past, trigeminal neuralgia was considered by the public as a sensory abnormality and was not taken seriously. However, with the advancement of technology and the continuous improvement of medical treatment, people are paying more attention to the pain sensation. However, there is a lack of reliable epidemiologic studies and risk factor analyzes for trigeminal neuralgia. The correlation with other diseases is also unclear. We aim to use Mendelian randomization analysis to explore some modifiable risk factors for the prevention, diagnosis, treatment, and long-term management of trigeminal neuralgia in the future. This will help in the early identification of patients with trigeminal neuralgia and reduce the chances of developing the disease with some positive psychological and behavioral interventions. And it will also inform epidemiologic studies of trigeminal neuralgia (9).

Mendelian randomization analysis is a method of exploring the causal relationship between exposure and outcome using genetic tools that avoid confounding bias and reverse causality (10). Mendelian randomization is similar to a randomized controlled trial, with high confidence in the efficacy of the statistical results. This method has been recognized as an effective tool for epidemiological statistical analysis, providing a reliable guide for the study of several diseases (11). We searched for 36 possible exposure factors based on a comprehensive search of previous studies. Mendelian randomization analysis was then applied to explore their causal relationship with trigeminal neuralgia.

## 2 Materials and methods

We obtained data from large genome-wide association analysis databases. Data was obtained through IEU OPENGWAS and FINNGEN(Ben (12, 13)). These data are publicly available for download and do not include individual-level data and therefore do not require additional ethical review. The design of this Mendelian randomization experiment follows the STROBE-MR statement (14).

### 2.1 Exposure

Due to the lack of previous relevant studies on risk factors for trigeminal neuralgia, to make the experiment as comprehensive as possible we performed a comprehensive search in PubMed. We searched for literature reviews, guidelines, meta-analyzes, and case reports on trigeminal neuralgia using the following keywords: trigeminal neuralgia, risk factors, association factors, case reports, lifestyle, cardiometabolic, diet, disease, and clinical examination. Previous articles exploring disease risk factors using Mendelian randomization analysis were referenced (15, 16). Information on references can be found in the Supplementary material. Thirty-six modifiable risk factors were eventually identified. They were categorized into 5 groups: Lifestyle(pack years of smoking, physical activity, sleep duration, Sleeplessness/insomnia, anxiety disorders, years of schooling, substance abuse, household income), Cardiometabolic (systolic blood pressure, diastolic blood pressure, triglycerides, HDL cholesterol, LDL cholesterol, total cholesterol, body fat percentage, omega-3 fatty acids, omega-6 fatty acids), Diet (alcohol consumption, coffee intake, processed meat intake, tea intake, fresh fruit intake), Disease (depression, malignant neoplasm of head and neck, cerebral aneurysm, type 2 diabetes, migraine, COVID-19, high blood pressure, autoimmune), Clinical examination (body mass index, fasting blood glucose, nerve growth factor, c-reactive protein level, estradiol levels, testosterone levels). All exposure information is displayed in the Table 1.

**Table 1:** Summary of modifiable risk factors for trigeminal neuralgia in the Mendelian randomization analysis.

Risk factors	SNPs	Sample size	Consortium or author	GWAS ID	F	Download link
Lifestyle						
Pack years of smoking	10	1,42,387	MRC-IEU	ukb-b-10831	77.56	https://gwas.mrcieu.ac.uk/datasets/ukb-b-10831/
Physical activity	18	3,77,234	Klimentidis YC	ebi-a-GCST006097	34.08	https://gwas.mrcieu.ac.uk/datasets/ebi-a-GCST006097/
Sleep duration	64	4,60,099	MRC-IEU	ukb-b-4424	42.32	https://gwas.mrcieu.ac.uk/datasets/ukb-b-4424/
Sleeplessness / insomnia	21	4,62,341	MRC-IEU	ukb-b-3957	49.54	https://gwas.mrcieu.ac.uk/datasets/ukb-b-3957/
Anxiety disorders	13	317,717	FINNGEN	NA	33.83	https://storage.googleapis.com/finngen-public-data-r9/summary_stats/finngen_R9_KRA_PSY_ANXIETY_EXMORE.gz
Years of schooling	295	7,66,345	SSGAC	ieu-a-1239	49.18	https://gwas.mrcieu.ac.uk/datasets/ieu-a-1239/
Substance abuse	9	300,720	FINNGEN	NA	35.52	https://storage.googleapis.com/finngen-public-data-r9/summary_stats/finngen_R9_KRA_PSY_SUBSTANCE_EXMORE.gz
household income	42	3,97,751	MRC-IEU	ukb-b-7408	41.51	https://gwas.mrcieu.ac.uk/datasets/ukb-b-7408/
Cardiometabolic						
systolic blood pressure	426	7,57,601	International Consortium of Blood Pressure	ieu-b-38	75.00	https://gwas.mrcieu.ac.uk/datasets/ieu-b-38/
diastolic blood pressure	289	7,57,601	International Consortium of Blood Pressure	ieu-b-39	75.88	https://gwas.mrcieu.ac.uk/datasets/ieu-b-39/
triglycerides	274	4,41,016	UK Biobank	ieu-b-111	149.40	https://gwas.mrcieu.ac.uk/datasets/ieu-b-111/
HDL cholesterol	308	4,03,943	UK Biobank	ieu-b-109	153.48	https://gwas.mrcieu.ac.uk/datasets/ieu-b-109/
LDL cholesterol	151	4,40,546	UK Biobank	ieu-b-110	184.50	https://gwas.mrcieu.ac.uk/datasets/ieu-b-110/
Total cholesterol	56	1,15,078	Borges CM	met-d-Total_C	96.66	https://gwas.mrcieu.ac.uk/datasets/met-d-Total_C/
Body fat percentage	18	4,54,633	MRC-IEU	ukb-b-8909	91.64	https://gwas.mrcieu.ac.uk/datasets/ukb-b-8909/
Omega-3 fatty acids	46	1,14,999	Borges CM	met-d-Omega_3	249.36	https://gwas.mrcieu.ac.uk/datasets/met-d-Omega_3/
Omega-6 fatty acids	50	1,14,999	Borges CM	met-d-Omega_6	116.17	https://gwas.mrcieu.ac.uk/datasets/met-d-Omega_6/
Diet						
Alcohol consumption	4	1,12,117	UK Biobank	ieu-a-1283	85.42	https://gwas.mrcieu.ac.uk/datasets/ieu-a-1283/
Coffee intake	38	4,28,860	MRC-IEU	ukb-b-5237	74.52	https://gwas.mrcieu.ac.uk/datasets/ukb-b-5237/
Processed meat intake	23	4,61,981	MRC-IEU	ukb-b-6324	38.54	https://gwas.mrcieu.ac.uk/datasets/ukb-b-6324/
Tea intake	40	4,47,485	MRC-IEU	ukb-b-6066	61.58	https://gwas.mrcieu.ac.uk/datasets/ukb-b-6066/
Fresh fruit intake	53	4,46,462	MRC-IEU	ukb-b-3881	45.66	https://gwas.mrcieu.ac.uk/datasets/ukb-b-3881/
Disease						
Depression	15	372,472	FINNGEN	NA	37.00	https://storage.googleapis.com/finngen-public-data-r9/summary_stats/finngen_R9_F5_DEPRESSIO.gz
Malignant neoplasm of head and neck	3	289,268	FINNGEN	NA	32.04	https://storage.googleapis.com/finngen-public-data-r9/summary_stats/finngen_R9_C3_HEAD_AND_NECK_EXALLC.gz
Cerebral aneurysm	4	1,95,203	Ishigaki K	bbj-a-96	42.87	https://gwas.mrcieu.ac.uk/datasets/bbj-a-96/
Type 2 diabetes	113	655,666	Xue A	ebi-a-GCST006867	76.34	https://gwas.mrcieu.ac.uk/datasets/ebi-a-GCST006867/
Migraine	3	306,314	FINNGEN	NA	42.15	https://storage.googleapis.com/finngen-public-data-r9/summary_stats/finngen_R9_G6_MIGRAINE.gz
COVID-19	4	18,87,658	COVID-19 Host Genetics Initiative	ebi-a-GCST011081	100.86	https://gwas.mrcieu.ac.uk/datasets/ebi-a-GCST011081/
High blood pressure	215	461,880	MRC-IEU	ukb-b-14177	62.27	https://gwas.mrcieu.ac.uk/datasets/ukb-b-14177/
Autoimmune	114	3,77,277	FINNGEN	NA	87.66	https://storage.googleapis.com/finngen-public-data-r9/summary_stats/finngen_R9_AUTOIMMUNE.gz
Clinical examination						
Body mass index	437	6,81,275	GIANT	ieu-b-40	74.60	https://gwas.mrcieu.ac.uk/datasets/ieu-b-40/
Fasting blood glucose	22	58,074	Manning AK	ebi-a-GCST005186	92.92	https://gwas.mrcieu.ac.uk/datasets/ebi-a-GCST005186/
Nerve growth factor	7	3,394	Folkersen L	prot-b-40	41.51	https://gwas.mrcieu.ac.uk/datasets/prot-b-40/
C-Reactive protein level	53	2,04,402	Ligthart, S	ieu-b-35	185.40	https://gwas.mrcieu.ac.uk/datasets/ieu-b-35/
Estradiol levels	2	1,63,985	Schmitz D	ebi-a-GCST90020092	38.36	https://gwas.mrcieu.ac.uk/datasets/ebi-a-GCST90020092/
Testosterone levels	149	4,25,097	Ruth KS	ebi-a-GCST90012114	85.73	https://gwas.mrcieu.ac.uk/datasets/ebi-a-GCST90012114/

### 2.2 Outcome

FINNGEN is a large public-private partnership aiming to collect and analyze genome and health data from 500,000 Finnish biobank participants. We used the latest GWAS summary data published on May 11, 2023, on trigeminal neuralgia from FINNGEN. It included 1,421 patients with trigeminal neuralgia and 301,193 controls. They were all of European origin.

### 2.3 Statistical analysis

Mendelian randomization analysis consists of three core assumptions: 1, that the selected instrumental variable SNP is strongly correlated with exposure. 2, that the instrumental variable SNP is uncorrelated with other confounders affecting the outcome. 3, and the instrumental variable SNP can only affect the outcome through exposure (17). To meet these assumptions, we set a value of *p*<=5e-08 for the instrumental variable SNP in the genome-wide association analysis to be significantly associated. Setting r2 < 0.001 in a length of 1,000 kb removes linkage disequilibrium (LD). The *F* value was calculated for each SNP, setting *F* > 10 to exclude weak instrumental bias (18). We used MRPRESSO to detect heterogeneity before performing Mendelian randomization analysis (19). And to eliminate outliers that can influence the direction judgment (20). After completing the data preparation, we used five common analysis methods including; Inverse variance weighted (IVW), MR Egger, weighted median, simple mode, and weighted mode. The inverse-variance weighted (IVW)method is the most efficient analysis method with valid instrumental variables, and so should generally be used as the primary analysis method. The basic idea of IVW is to first calculate the causal effect estimates for each SNP, and then utilize the fixed effect model of Meta-analysis to combine the causal effect estimates for each instrumental variable to obtain a total causal effect estimate. The model combined the causal effect estimates for each instrumental variable to obtain a total causal effect estimate (21, 22). After completing the analysis, we performed sensitivity tests to determine if there were some violations of the MR assumptions and potential bias. Sensitivity tests, including heterogeneity tests, multiple validity tests, and leave-one-out tests were performed after each analysis to test the reliability of the results. Two-tailed tests were taken for all tests. Since they were dichotomous variables, we eventually converted to ORs to visualize the relationship between exposure and outcome more visually. In summary, all our experimental designs and numerical choices were performed concerning the recommendations in the STROBE-MR statement. All the statistical analyzes were performed in R 4.3.1 with the TwoSampleMR 0.5.6 package.

## 3 Results

**Figure 1:**
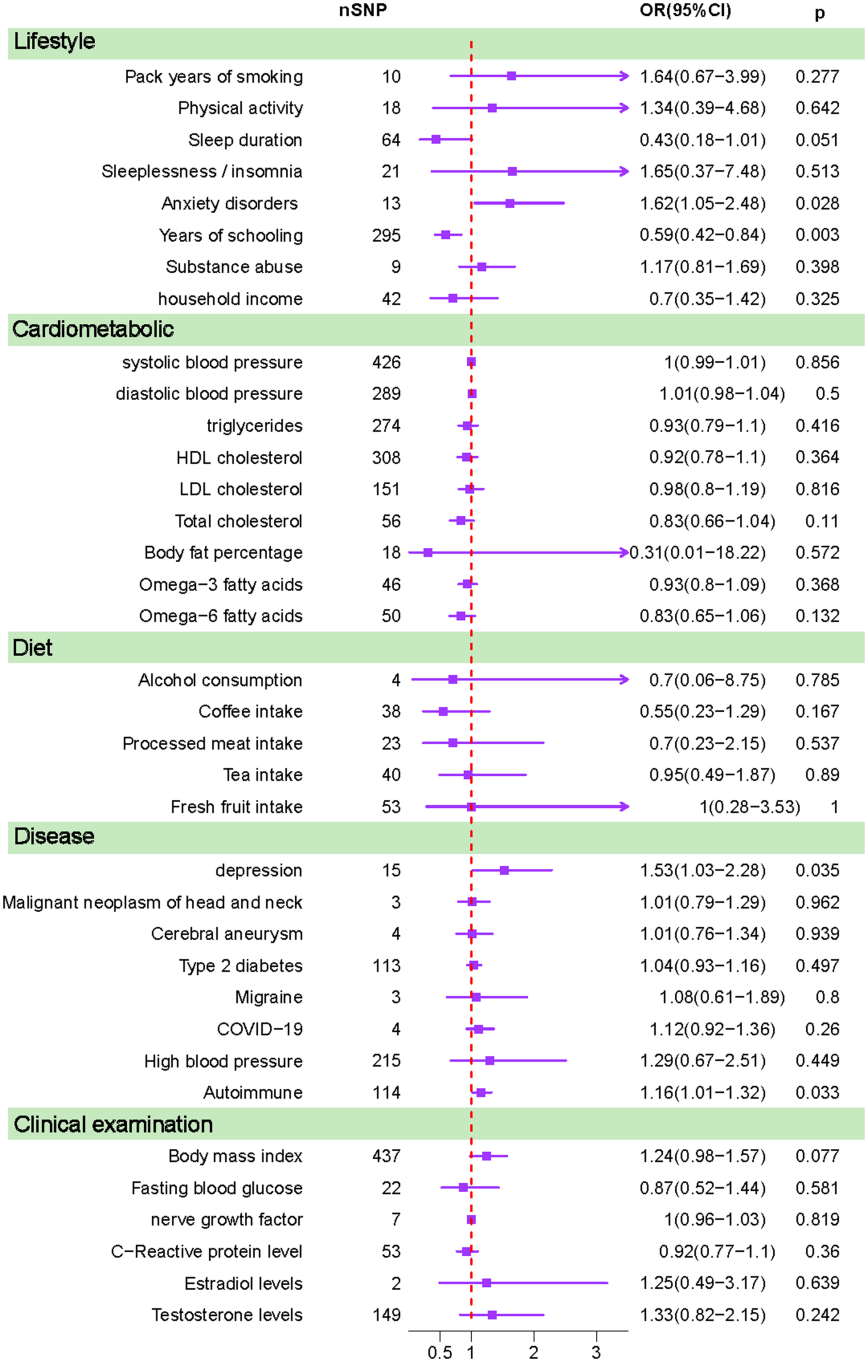
Results calculated using inverse-variance weighted method (IVW).

We included a total of 36 modifiable risk factors. All the project information is summarized in Table 1. Five common methods were used in conducting the Mendelian randomization analysis, with the results of IVW ultimately serving as the primary basis for judgment. In Figure 1 the results of the IVW method are shown. Please refer to the Supplementary material for a summary of the other results. We found Years of schooling [OR (95%CI), 0.59(0.42–0.84), *p* = 0.003] to be a significant protective factor. Anxiety disorders [OR (95%CI), 1.62(1.05–2.48), *p* = 0.028], Depression [OR (95%CI), 1.53(1.03–2.28), *p* = 0.035] and Autoimmune [OR (95%CI), 1.16(1.01–1.32), *p* = 0.033] were significant risk factors. Sleep duration [OR (95%CI), 0.43(0.18–1.01), *p* = 0.051] was a close protective factor. Body mass index [OR (95%CI), 1.24(0.98–1.57), *p* = 0.077] was a close risk factor.

## 4 Discussion

Studies based on European populations have found a lifetime prevalence of trigeminal neuralgia of 0.16–0.3%, with an incidence of 12.6–27.0/100,000 person-years. For reasons that are not yet clear, trigeminal neuralgia affects more women (60%) than men (40%). The average age of onset is reported to be 53–57 years (9). The pain tends to get progressively worse if the patient is not treated. Some patients are even unable to sleep or eat because of the pain. However, there is a lack of reliable epidemiologic studies on risk factors for trigeminal neuralgia. Mendelian randomization as a new epidemiological research tool has already played an important role in the exploration of risk factors for a variety of diseases. Therefore, we propose for the first time to use this tool to study trigeminal neuralgia, an underappreciated neurological disease.

We found that Years of schooling and Sleep duration may be protective against trigeminal neuralgia. Years of schooling are even more statistically significant. This suggests that the longer one receives education the less likely one is to develop trigeminal neuralgia. We believe this may be because populations with more time in education have a stronger desire for healthcare and personal wellness. This is, of course, consistent with previous observations that the incidence of trigeminal neuralgia gradually increases with age (1). The same result has been found in several other MR articles. And the length and extent of education are a possible protective factor in a variety of other diseases (23–26). Sleep duration is a suggested protective factor. Chronic sleep deprivation can be extremely stressful both physically and psychologically. Many previous studies have also revealed a link between sleep duration and a variety of diseases such as anxiety disorders, depression, cardiovascular disease (CVD) coronary heart disease (CHD), lung cancer, and stroke (27–31). And as our results show anxiety disorders and depression are risk factors for trigeminal neuralgia.

Anxiety disorders and Depression are considered risk factors. And in the clinic, we find that many patients show abnormal mental status because of chronic pain. And while the patient’s pain is relieved after treatment, anxiety, and depression are still areas of concern (32, 33). Long-term pain medication use may also cause a variety of adverse effects. Studies have shown that long-term opioid use may also increase Anxiety disorders and Depression (34). Therefore, more attention should be paid to the psychological counseling of patients with trigeminal neuralgia rather than simply prescribing painkillers in the treatment and long-term guidance of patients with trigeminal neuralgia (35). Based on our clinical experience, we have found that patients with trigeminal neuralgia often have late nights, chronic sleep deprivation, anxiety, and depression, especially women, and such patients, after microvascular decompression therapy, have a greater proportion of delayed healing. Therefore, during the treatment of trigeminal neuralgia patients, we should not only focus on surgery or other treatments, but also focus on the psychological counseling and health education of patients.

Autoimmune is recognized as a risk factor. Several atypical cases of trigeminal neuralgia have appeared in previous literature. They do not produce symptoms due to compression but due to the primary disease. Reports have included multiple sclerosis, systemic lupus erythematosus, diffuse large B-cell lymphoma, and idiopathic inflammatory and dry syndromes, among others (36–41). This suggests a possible role for immune factors in trigeminal neuralgia. Infiltrating macrophages and lymphocytes were found to be increased in the trigeminal neuralgia rat model. This suggests that neuroimmune cells may be involved in the pathogenesis of the TN rat model (42). Of course the mechanisms need to be further studied.

Body mass index as a possible risk factor. BMI is widely used as an indicator to assess body fatness, and its association with a variety of diseases is well recognized. A high body mass index may contribute to a variety of chronic diseases (43). Physicians should emphasize this in both patient health education and perioperative management.

To the best of our knowledge, the cause of trigeminal nerve development remains a mystery. Both autopsy and clinical studies have found that neurovascular compression(NVC) is not 100% present in trigeminal neuralgia (44–46). Neurovascular compression has also been found in patients with non-trigeminal neuralgia (47). In one study, it was found that NVC was not detected by surgical exploration in 9% of patients, and imaging showed no vascularity associated with the nerve in 12% of patients (48). In the work of clinicians, patients with trigeminal neuralgia are not found to have peripheral vascular compression, but still present with trigeminal neuralgia. The emergence of this phenomenon has led to active investigation by scholars. We hope that this will help to provide some basis for early diagnosis and prevention of trigeminal neuralgia and provide ideas for complex mechanistic studies later on.

We are the first to apply Mendelian randomization analysis as a method to analyze the factors contributing to the pathogenesis of trigeminal neuralgia. Although the Mendelian randomization method has the advantages of being similar to randomized control and avoiding reverse causality. But it also has some limitations. For example: the data sources for the study were all European populations may not be representative of other populations. We only analyzed some known factors and those unknown factors may also play an important role in trigeminal neuralgia. For some results although not statistically significant, this may be due to low statistical power. Risk factors for trigeminal neuralgia were searched and screened as much as possible in the design of the experiment, but not all of them had corresponding GWAS data. We also found that the GWAS data for some risk factors did not meet the requirements of Mendelian randomization analysis during the analysis. So, we had to shed some possible risk factors. Thus, our analysis may have some limitations. However, our results are useful for screening the high prevalence of trigeminal neuralgia and have the potential to reduce the incidence of trigeminal nerves through positive psychological and lifestyle interventions. It provides ideas for subsequent mechanistic studies and clinical management of trigeminal neuralgia.

## 5 Conclusion

In summary, we are the first to use genetic tools to study trigeminal neuralgia risk factors. We used Mendelian randomization analysis, an emerging epidemiological research tool, to explore some of the modifiable risk factors for trigeminal neuralgia. Mendelian randomization analysis shows Years of schooling and Sleep duration as protective factors. Anxiety disorders, Depression, Autoimmune, and Body mass index are risk factors. This will help in the research of diagnosis, treatment, and mechanism of trigeminal neuralgia. And reduce the prevalence of trigeminal neuralgia through positive psychological and lifestyle interventions.

## Data availability statement

The original contributions presented in the study are included in the article/Supplementary material, further inquiries can be directed to the corresponding authors.

## Author contributions

XW: Data curation, Formal analysis, Methodology, Software, Writing – original draft, Writing – review & editing. HZ: Data curation, Formal analysis, Investigation, Writing – original draft. SZ: Formal analysis, Writing – review & editing. XH: Writing – review & editing, Conceptualization, Investigation. ZW: Funding acquisition, Resources, Writing – review & editing. YL: Funding acquisition, Resources, Writing – review & editing.
